# The complete mitochondrial genome of Kuhl’s pipistrelle, *Pipistrellus kuhlii* (Chiroptera: Vespertilionidae)

**DOI:** 10.1080/23802359.2016.1176886

**Published:** 2016-06-21

**Authors:** Andrea G. Locatelli, David Jebb, Emma C. Teeling

**Affiliations:** School of Biology and Environmental Science, University College Dublin, Dublin, Ireland

**Keywords:** Chiroptera, mitogenome, mtDNA, *Pipistrellus kuhlii*, Vespertilionidae

## Abstract

The Kuhl’s pipistrelle (*Pipistrellus kuhlii*) is a small, vespertilionid bat species, with a large range extending from the Iberian Peninsula into the Near East and the Arabian Peninsula. In this study, we determine for the first time the complete mitogenome of this species. The *P. kuhlii* mitogenome is 16,991 base pairs long with 37 genes and 1 control region, showing conserved gene content and order with other vertebrate mitogenomes. The length of the 22 tRNA genes ranges between 60 bp (tRNA-Ser) and 75 bp (tRNA-Leu). The D-loop region is 1553 bp long with low CG content (39.8%).

Kuhl’s pipistrelle (*Pipistrellus kuhlii*) is a small, vespertilionid bat species (5–7g), with a geographic distribution that extends from Mediterranean Europe into Western India (Molur & Walker 2003). A recent study based on a combination of mitochondrial and nuclear markers, suggests that there are four *P. kuhlii* sub-populations identified as the Atlantic Islands lineage, the Western lineage, the Eastern lineage and the Middle Eastern lineage (Andriollo et al. 2015).

*P. kuhlii* is commonly found in anthropic environments such as agricultural and urban areas. The species can also be found in diverse environments from deserts to temperate grasslands and at altitudes up to 2000 metres (Aulagnier et al. 2008). Breeding occurs in August and September and females give birth to a single pup each year (Mills & Hes 1997; Qumsiyeh 1996). The species is classified as ‘Least Concern’ on the IUCN red list of threatened species (Aulagnier et al. 2008).

*P. kuhlii* was captured and identified in the field by A. G. Locatelli in northern Italy (Cavallermaggiore [CN] N44°41′; E7°42′). The DNA was extracted from wing biopsies (3 mm diameter) using the DNeasy blood and tissue kit (Qiagen Inc., Cat. No. 69506) from sample PKU003 (Teeling DNA Repository, UCD). Two primers sets, as detailed in Jebb et al. (2015), were used to amplify the whole mtDNA in two partially overlapping fragments with the Expand^TM^ Long Range dNTPack (Cat. No. 04829034001, Sigma-Aldrich, Chicago, IL). Amplicons were pooled in equimolar amounts and sequencing libraries prepared with the Nextera XT Library preparation Kit (Cat. No. FC-131-1024, Illumina). Libraries were sequenced using the MiSeq Illumina 2000 platform with a 250 bp paired-end reads. A *de novo* assembly was performed with ABySS (Simpson et al. 2009). Annotations were inferred using ARWEN v1.2 and Geneious 7.0.6 with *Pipistrellus abramus* (AB061528) as a reference (Laslett & Canbäck 2008; Kearse et al. 2012).

*P. kuhlii* mitogenome (GenBank Accession Number: KU058655) contains 13 protein-coding genes, 22 tRNA genes, 2 rRNA, 1 D-loop similar to other bat species. H-strand base composition is 32.4% A, 28.5% T, 24.9% C and 14.2 G%. The ND1, ND5 and ND6 genes start with the ATA codon, ND2 and ND3 with ATT while all others protein-coding genes start with ATG. All tRNAs are predicted to produce the canonical cloverleaf structure, except for the tRNA-Ser which lacks the dihydrouridine loop, and range from 60 (tRNA-Ser) to 75 (tRNA-Leu) bp in length. A phylogenetic tree of the relationships between 19 Chiroptera inferred from complete mitogenome sequences is shown in Figure 1. The tree was constructed with MrBayes 3.2.6 (Huelsenbeck & Ronquist 2001). The alignment was made with MAFFT and filtered with Gblocks (Castresana 2000). Based on the AIC, the GTR + I + Γ nucleotide substitution model with four rate categories was chosen using JModelTest 2 (Darriba et al. 2012). Chains were run for 1,100,000 generations and sampled every 200. The first 110,000 were discarded as burn-in. *P. kuhlii* clustered with *Nyctalus noctula* and *P. abramus*, with *P. kuhlii* and *N. noctula* as sister taxa as previously reported (Hoofer & van Den Bussche. 2003; Hulva et al. 2004).

**Figure 1.**
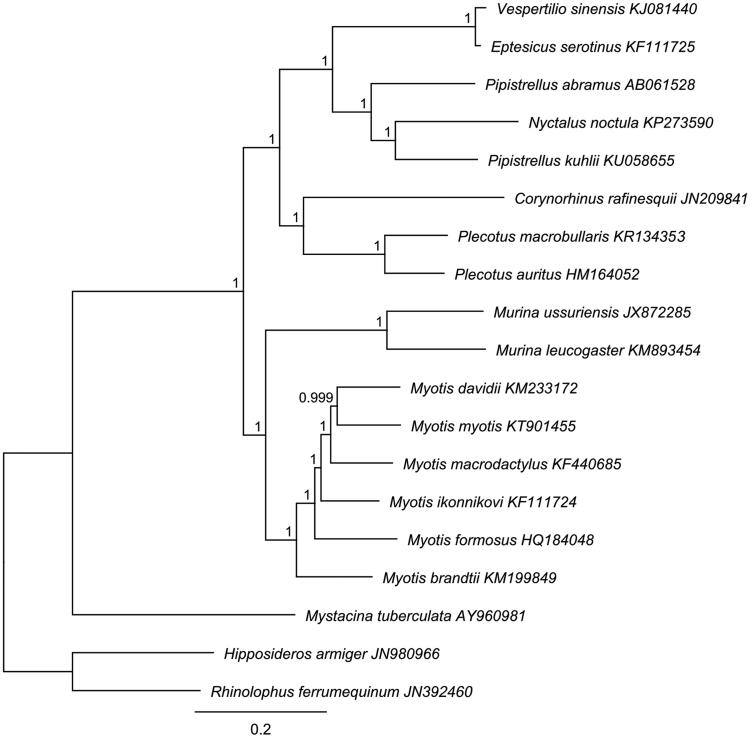
Phylogenetic tree built using Bayesian Inference analysis of 19 bats complete mitogenome sequences. Two yinpterochiropteran species (*Rhinolophus ferrumequinum* and *Hipposideros armiger*) were used as outgroups. Values at nodes indicate posterior probabilities. GenBank accession numbers are reported next to each species name.
